# Small Interfering RNA Strategies to Overcome Drug Resistance in Cancer: Pathway Targeting and Translational Advances

**DOI:** 10.5152/eurasianjmed.2026.251350

**Published:** 2026-02-18

**Authors:** Sanaa K. Bardaweel, Rima Hajjo, Dima A. Sabbah, Daliah Al Qasem

**Affiliations:** 1 Department of Pharmaceutical Sciences, The University of Jordan School of Pharmacy, Amman, Jordan; 2 Department of Pharmacy, Al-Zaytoonah University of Jordan Faculty of Pharmacy, Amman, Jordan; 3 Laboratory for Molecular Modeling, Division of Chemical Biology and Medicinal Chemistry, The University of North Carolina Eshelman School of Pharmacy, Chapel Hill, USA

**Keywords:** Cancer therapy, gene silencing, resistance, siRNA

## Abstract

The emergence of therapeutic resistance remains the primary challenge to achieving durable responses in cancer treatment, thereby limiting the long-term efficacy of several chemotherapies and targeted agents. Resistance may develop through diverse and often overlapping mechanisms. Conventional approaches to overcome resistance, such as dose escalation or combination regimens, are frequently constrained by systemic toxicity and adaptive tumor evolution. Moreover, many central drivers of resistance, including transcription factors and non-enzymatic regulatory proteins, remain inaccessible to therapeutics. Small interfering RNAs (siRNAs) offer a fundamentally distinct and pathway-centric approach to overcoming drug resistance by enabling sequence-specific degradation of resistance-associated messenger RNAs. By acting upstream of protein synthesis, siRNA therapeutics can directly suppress both druggable and traditionally undruggable targets, including efflux transporters, anti-apoptotic regulators, DNA repair enzymes, epigenetic modifiers, epithelial-mesenchymal transition transcription factors, and key oncogenic drivers such as KRAS and MYC. Advances in chemical modification and delivery platforms have significantly improved siRNA stability, specificity, and pharmacokinetic durability, enabling sustained gene silencing *in vivo*. The current review examines the molecular principles of siRNA-induced gene silencing and evaluates the current siRNA-based strategies targeting key mechanisms of chemotherapy resistance. Preclinical and translational studies demonstrating the reversal of multidrug resistance, restoration of apoptotic sensitivity, disruption of DNA repair–mediated tolerance, and suppression of adaptive oncogenic signaling are highlighted. In addition, the emerging clinical trials that validate siRNA-based approaches in cancer treatment are summerized.

**Main Points**

- Therapeutic resistance is a major limitation of cancer treatment, arising from complex and adaptive molecular mechanisms.
- Small interfering RNA (siRNA) therapeutics offer a pathway-centric strategy to directly silence resistance drivers, including traditionally undruggable targets.
- Advances in siRNA design and delivery have enabled stable, specific, and durable gene silencing *in vivo*.
- Preclinical and early clinical studies show that siRNA-based approaches can reverse drug resistance and restore treatment sensitivity.

## Introduction

Despite the significant progression in cancer therapy, the emergence of drug resistance remains the primary hurdle to achieving long-term remission and cure.^1^ Multidrug resistance is generally classified into 2 types: intrinsic (pre-existing non-responsiveness) or acquired (mechanisms developed during therapy).^2^ Underlying mechanisms may include the activation of cellular survival pathways, such as anti-apoptosis and epithelial-to-mesenchymal transition, in addition to the modification of drug pharmacokinetics through enhanced drug efflux by transporters or enzymatic inactivation, preventing the drug from reaching its target.^2^ Such routes are often led by genetic mutations, epigenetic changes, and the tumor microenvironment, enabling the cancer cell to bypass the drug’s cytotoxicity.^3^

Available strategies, such as combination chemotherapy and the use of high-dose regimens, are often limited by high systemic toxicity. Additionally, the current targeted therapies aim to bypass specific resistance routes, which frequently result in the rapid emergence of new resistance mechanisms. A fundamental limitation is that traditional small-molecule inhibitors and antibodies are restricted to targeting proteins that have accessible binding pockets.^4^ This constraint leaves a vast segment of the cancer proteome, including critical transcription factors and non-enzymatic effectors that drive resistance pathways, functionally “undruggable.” Therefore, there is an urgent need for therapeutic modalities that can limit disease progression at the genetic level, overcoming these structural and biochemical restraints.

RNA interference (RNAi), a post-transcriptional gene silencing process that occurs naturally in cells, offers a potent and orthogonal strategy to the development of drug resistance.^5^ Thesmall interfering RNA (siRNA) guides the cleavage of complementary target messenger RNA (mRNA) by the RNA-induced silencing complex (RISC).^6^ By specifically targeting the mRNA that encodes crucial factors for resistance, siRNA may provide a pathway-centric approach for overcoming drug resistance to currently available chemotherapies or targeted agents.^6^ The utility of siRNA is rooted in several inherent advantages: high specificity derived from base-pairing complementarity, which results in less non-specific or off-target effects; excessive modularity and flexibility to target virtually any gene to allow rapid tailoring to specific resistance mechanisms;^6^ and remarkably, the ability to target the undruggable genome by removing the function of regulatory proteins at the transcript level.

This review explores the various applications of siRNA in cancer treatment, focusing on its ability to silence key resistance pathways and the latest translational advances in clinical settings. The current strategies for pathway targeting, recent clinical developments, and the remaining hurdles for integrating siRNA-based therapies into standard oncology practice are discussed.

### Principles of Small Interfering RNA–Induced Gene Silencing

Small interfering RNA mediates gene silencing through the evolutionarily conserved RNAi pathway, enabling highly sequence-specific suppression of gene expression at the post-transcriptional level.^7^ These are short double-stranded RNA molecules, typically 21-23 nucleotides in length, with characteristic 2-nucleotide 3′ overhangs.^7^ In mammalian cells, therapeutic and experimental siRNAs are usually delivered as synthetic duplexes that bypass the Dicer processing while retaining full compatibility with the endogenous RNAi machinery.

Following cytoplasmic entry, the siRNA duplex is loaded into the RISC, a multiprotein assembly centered on Argonaute (Ago) proteins.^8^During RISC maturation, the passenger strand is removed, either through Ago2-mediated cleavage or unwinding. On the other hand, the guide strand is selectively retained based on thermodynamic asymmetry at its 5′ end.^8^ This guide strand then serves as a sequence-specific template that directs RISC to complementary mRNA targets.

Target recognition requires almost perfect complementarity between the guide strand and the target mRNA. Upon binding, Ago2, which is the only catalytically active Argonaute in mammals, cleaves the mRNA at a precise site corresponding to nucleotides 10-11 of the guide strand. The cleaved mRNA fragments are subsequently degraded by cellular exonucleases, which results in rapid depletion of the transcript and suppression of protein synthesis. Because RISC can be recycled after each cleavage event, a single siRNA molecule can mediate multiple rounds of target mRNA degradation, conferring potent and durable gene silencing.^8^

Although siRNA activity is highly specific, off-target effects can arise through partial complementarity between the siRNA seed region (nucleotides 2-8) and unintended transcripts, a mechanism analogous to microRNA-mediated repression.^9^ Consequently, careful sequence selection and chemical modification are essential for therapeutic application.

Notably, native RNA is inherently unstable in biological fluids and can activate innate immune responses. To overcome these limitations, therapeutic siRNAs incorporate chemical modifications such as 2′-O-methyl, 2′-fluoro, and phosphorothioate linkages, which enhance nuclease resistance, reduce immunogenicity, and improve pharmacokinetic properties without impairing RISC loading or silencing efficacy.^10^

Together, these molecular principles underpin the utility of siRNA as a precise and adaptable platform for silencing disease-associated genes, including targets that are inaccessible to conventional small-molecule or antibody-based therapies.

### Small Interfering RNA–Targetable Molecular Mechanisms of Chemotherapy Resistance in Cancer Cells

**Figure 1.**
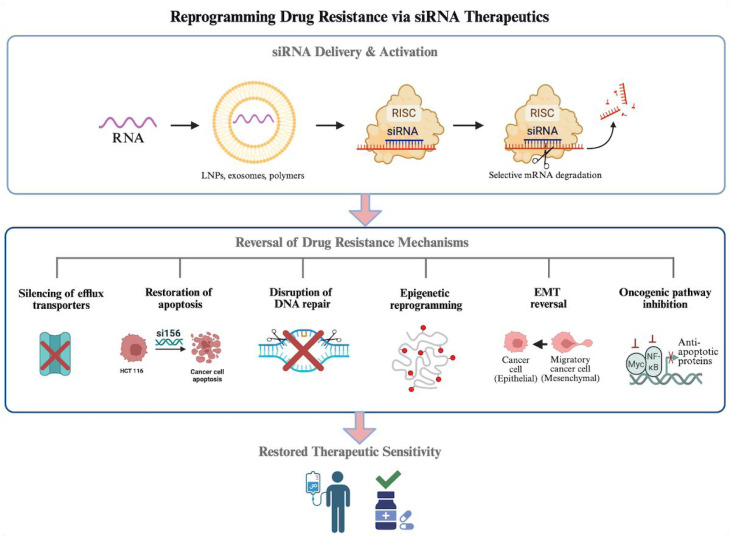
Mechanisms by which siRNA overcomes multi-layered drug resistance in cancer. siRNA delivered via lipid nanoparticles or extracellular vesicles enters tumor cells and is incorporated into the RNA-induced silencing complex (RISC), leading to sequence-specific degradation of target mRNAs. This results in suppression of efflux transporters, restoration of apoptotic pathways, disruption of DNA repair, inhibition of epigenetic drivers of transcriptional resistance, reversal of epithelial–mesenchymal transition (EMT), and attenuation of oncogenic signaling pathways, collectively enhancing or restoring therapeutic sensitivity. *Figure generated by Biorender.*

Chemotherapy resistance remains one of the most formidable barriers to durable cancer treatment, arising from a complex and adaptive network of molecular alterations that enable tumor cells to evade cytotoxic stress. These resistance mechanisms operate at multiple regulatory levels, including drug transport, apoptotic signaling, DNA damage repair, epigenetic remodeling, epithelial–mesenchymal transition (EMT), and oncogenic pathway activation. Together, they form an interconnected survival architecture that allows malignant cells to dynamically reprogram their phenotype in response to therapeutic pressure. Importantly, many of these resistance pathways are driven by aberrant gene expression rather than irreversible genetic mutations, rendering them particularly amenable to therapeutic modulation. However, siRNA has emerged as a powerful strategy to selectively silence resistance-associated genes with high specificity, enabling direct intervention at the transcriptomic level, as illustrated in Figure 1.

#### Small Interfering RNA–Mediated Silencing of Drug Efflux Transporters: Silencing the Molecular Bouncers

Chemotherapy resistance occurs due to several mechanisms, including alterations in the drug efflux or its influx inside the tumor cell. The ATP-binding cassette (ABC) transporters function as molecular efflux pumps embedded in the cell membrane.^11^ When overexpressed, specific transporters, such as P-glycoprotein (P-gp/ABCB1), multidrug resistance–associated protein 1 (MRP1/ABCC1), and breast cancer resistance protein (BCRP/ABCG2), act like powerful bouncers, recognizing a wide range of structurally diverse chemotherapeutic agents and pumping them out of the cell.^12^ On the other hand, the reduced folate carrier (RFC) is responsible for the uptake of methotrexate (MTX), a dihydrofolate reductase (DHFR) inhibitor.^13^ Tumors with RFC-inactivating mutations have demonstrated increased resistance to MTX.^13^ This delicate balance between efflux and influx systems dictates the effective concentration of the drug at its intracellular target, making transport proteins central to resistance mechanisms.

Multidrug resistance (MDR) in breast cancer can be effectively reversed using pH-sensitive carbonate apatite nanoparticles to co-deliver siRNAs targeting the mRNA of ABCB1 (P-gp) and ABCG2 (BCRP). These inorganic nanocarriers facilitate efficient cellular entry and escape the endosome-lysosome system by rapidly dissolving in acidic pH, releasing the siRNA payload directly into the cytoplasm.^14^ In resistant MCF-7 cell lines, this targeted silencing dose-dependently restores sensitivity to traditional chemotherapeutic agents such as doxorubicin, paclitaxel, and cisplatin.^14^ Notably, the simultaneous delivery of multiple siRNAs targeting both transporters produces a more robust increase in chemosensitivity than single-target approaches, highlighting the potential of this delivery concept for overcoming complex MDR phenotypes in clinical settings.^14^ Beyond ABCB1, recent evidence underscores the critical role of other transporters, such as ABCC11, in conferring resistance to diverse agents like eribulin, paclitaxel, and doxorubicin across various breast cancer subtypes.^15^ Similarly, in osteosarcoma, the overexpression of ABCB1 and ABCC1 has been directly linked to MDR.^16^ Targeted silencing of these transporters, often through lipid-modified or chitosan-based delivery platforms, has proven to be a robust strategy for restoring chemosensitivity and enhancing the synergistic effects of traditional chemical drugs.

In addition to ABC transporters, the ATPase copper transporting alpha (ATP7A) significantly contributes to chemotherapy resistance, particularly in colorectal cancer (CRC).^17^This transporter facilitates the efflux of platinum-based agents like oxaliplatin, sequestering the drug to prevent it from reaching its nuclear targets. Recent research indicates that silencing ATP7A through siRNA-mediated knockdown is a novel and effective strategy for overcoming platinum resistance in CRC models.^17^

#### Small Interfering RNA–Driven Suppression of Anti-Apoptotic Signaling: Overcoming Evasion of the Kill Switch

The evasion of apoptosis pathways is a recognized hallmark of cancer that contributes significantly to both tumorigenesis and drug resistance, effectively blocking programmed cell death by disrupting the molecular balance between survival and execution proteins.^18^ This resistance is achieved when the vital process mediated by caspases, the key executioners of apoptosis, is interrupted.^18^ The BCL-2 protein family acts as the central gatekeeper, where the overexpression of anti-apoptotic members (like BCL-2 and BCL-xL) creates a protective firewall, physically blocking pro-apoptotic proteins from permeabilizing the mitochondrial membrane and initiating the caspase cascade.^19^ Furthermore, the tumor cell uses endogenous suppressors, namely the inhibitors of apoptosis proteins (IAPs), with XIAP serving as a dominant negative regulator that directly binds to and inhibits active caspase-9 and caspase-3/7.^20^ Finally, this intrinsic resistance is complemented by external signals, as the hyperactivation of survival cascades, such as the (phosphatidylinositol 3-kinase (PI3K)/protein kinase B (AKT)/mechanistic target of rapamycin (mTOR) signaling pathway) PI3K/Akt/mTOR and MAPK/ERK pathways, guarantees resilience; these cascades provide compensatory growth signals and simultaneously suppress apoptosis by modulating BCL-2 family proteins, ensuring the tumor evades the therapeutic “kill” signal.^21^

To overcome the evasion of programmed cell death, siRNA has emerged as a high-precision tool for silencing the “firewall” of anti-apoptotic proteins, effectively restoring the cellular “kill switch” in resistant tumors. Research by Rieger et al^22^ and Tanaka et al^23^ has demonstrated that targeting overexpressed apoptosis inhibitors, specifically Bcl-xL in bladder cancer and survivin in MCF-7 breast cancer, exerts potent anti-proliferative effects and restores sensitivity to cisplatin and paclitaxel, respectively.^22^^,^^23^ By degrading the mRNA of these survival proteins, siRNA disrupts the protective molecular balance, allowing pro-apoptotic signals to proceed through the mitochondrial membrane and initiate the Caspase cascade.

Recent therapeutic strategies emphasize the co-delivery of siRNA with conventional chemotherapy or chemical inhibitors to achieve synergistic tumor suppression. Utilizing diverse nanoparticle platforms, studies have successfully combined Bcl-2 siRNA with agents such as doxorubicin, curcumin, or temozolomide, as well as survivin siRNA with etoposide.^24^ These combinatorial approaches consistently result in enhanced cellular uptake, significant knockdown of BCL-2 and survivin, inhibited tumor growth, and a robust induction of apoptosis.

### Small Interfering RNA–Induced Disruption of DNA Damage Repair: Disabling the Self-Healing System

Tumor cells often achieve chemoresistance by becoming highly efficient at repairing drug-induced genetic damage, thereby neutralizing the intended cytotoxic effects of many conventional therapies. Many chemotherapeutic agents and radiation treatments kill cancer cells by causing catastrophic damage to their DNA, such as double-strand breaks or inter-strand cross-links. Resistant cells, however, become “molecular mechanics,” upregulating components of the DNA Damage Repair (DDR) pathways to fix this damage rapidly.^25^ Key players in this resistance mechanism include the poly-ADP-ribose polymerase family and the central signaling kinases Ataxia Telangiectasia Mutated (ATM) and ATR (ATM and Rad3 related).^26^ These elements act as damage sensors and coordinators, rapidly recruiting repair proteins like BRCA1/2 to mend broken DNA strands.^27^ By enhancing the capacity and speed of these DDR pathways, tumor cells minimize the lethal accumulation of genetic damage, allowing them to withstand doses of chemotherapy that should be curative and resume proliferation.^27^

To disable the “self-healing” capabilities of resistant tumors, siRNA-mediated silencing is employed to inhibit key sensors and effectors within the DDR pathways. By degrading the mRNA of these “molecular mechanics,” siRNA prevents the recruitment of repair proteins like BRCA1/2, ensuring that drug-induced double-strand breaks lead to permanent cell cycle arrest or apoptosis rather than cellular recovery.^27^ This approach often aims to achieve synthetic lethality, where the simultaneous presence of drug-induced damage and siRNA-inhibited repair leaves the cancer cell with no viable survival pathway. Recent studies have highlighted the clinical significance of targeting BARD1 and BRCA1, which are highly expressed in tamoxifen-resistant breast cancers that also exhibit cisplatin resistance; their silencing has been shown to successfully re-sensitize these tumors to cisplatin treatment both *in vitro* and *in vivo*.^28^ Similarly, Wang et al^29^ discovered that the overexpression of ERCC1 and BRCA1 in drug-resistant lung cancer cells serves as a primary survival mechanism; however, silencing these genes with siRNA effectively inhibits cell proliferation, induces apoptosis, and restores chemosensitivity.

#### Small Interfering RNA Targeting of Epigenetic Regulators Driving Transcriptional Resistance

Epigenetic modifications represent a critical, non-genetic layer of control over gene expression that tumor cells exploit to drive resistance.^30^ These modifications, which include DNA methylation, histone modifications, and non-coding RNAs, do not alter the underlying DNA sequence but profoundly change the gene transcription profile.^30^ Histone modifications involve specialized binding structures such as chromo- (methylation) and bromo- (acetylation) domains, while large protein complexes, including SWI/SNF, ISWI, and CHD remodelers, actively change chromatin structure to regulate accessibility.^30^ The enzymes governing these changes (DNA- and histone-modifying enzymes) are frequently mutated and contribute significantly to tumorigenesis, ultimately establishing a unique tumor epigenome that favors survival in the presence of therapy.^30^ Crucially, this mechanism contributes to reversible non-genetic tumor heterogeneity, meaning the drug-resistant state can be rapidly induced or reversed without permanent DNA mutation, often resulting in the transcriptional silencing of pro-apoptotic genes or the activation of drug transporters.^31^ Because these epigenetic marks are inherently reversible, they offer a potent therapeutic opportunity: combining traditional cytotoxic or targeted drugs with epigenetic modifiers, such as HDAC inhibitors, DNMT inhibitors, or BET inhibitors, can reverse the resistant epigenome and re-sensitize tumor cells to treatment.^31^

Given that epigenetic dysregulation underlies reversible transcriptional programs associated with drug tolerance and resistance in cancer, siRNA offers a highly specific tool to intervene at the level of epigenetic regulators themselves. Aberrant expression or activity of DNA methyltransferases (DNMTs), histone modifiers (e.g., histone demethylases/methyltransferases), and chromatin remodelers contribute to transcriptional repression of tumor suppressors or activation of survival pathways that enable therapy evasion. Targeting these regulators with siRNA can directly reverse epigenetic repression and resensitize resistant tumor cells to therapy.^32^

One major approach uses siRNA to knock down key “writers” and “erasers” of epigenetic marks. For example, siRNA against DNMT1 or other DNMT family members has been shown in preclinical models to reduce aberrant promoter methylation and reactivate silenced tumor suppressor genes, thereby altering gene expression profiles linked to resistance phenotypes.^32^ Similarly, siRNA targeting histone-modifying enzymes, such as histone demethylases and methyltransferases, can remodel chromatin marks associated with transcriptional repression. Preclinical studies have used siRNA to suppress EZH2, a histone methyltransferase that drives H3K27 trimethylation at tumor suppressor loci, and LSD1 family demethylases that contribute to oncogenic transcriptional programs and resistance.^33^

#### Small Interfering RNA–Mediated Reversal of Epithelial–Mesenchymal Transition (EMT)–Associated Drug Resistance: The Cancer Cell’s Shape-Shifting Defense

The epithelial–mesenchymal transition is a profound biological program where cancer cells undergo a “shape-shift,” transforming from organized, static epithelial cells into mobile, invasive mesenchymal cells, a process tightly linked to chemoresistance and metastasis.^34^Core transcription factors, notably Snail, Twist, and ZEB1, drive this transition by repressing epithelial markers while activating mesenchymal genes, enabling the cell to survive therapeutic stress.^34^ The resulting mesenchymal phenotype grants cells increased motility, stemness, and powerful defense mechanisms against chemotherapy.^34^ Furthermore, the EMT process deeply affects the tumor microenvironment: transformed cells secrete enzymes such as matrix metalloproteinases, which degrade the extracellular matrix. This degradation not only facilitates invasion and metastasis but also generates signals that actively promote drug resistance, creating a protective niche that shields the transformed tumor cell from the cytotoxic effects of anticancer agents.^35^

To combat the “shape-shifting” defense of cancer cells, siRNA-mediated silencing is utilized to reverse the EMT and effectively reprogram invasive cells back into a drug-sensitive epithelial state. Research across various malignancies, including glioblastoma, hypopharyngeal, breast, prostate, gastric, lung, hepatic, and colon cancers, has demonstrated that silencing Snail, ZEB1, ZEB2, MMP2, and MMP9 can successfully overcome chemotherapy resistance.^36^ These studies highlight that gene knockdown reverses the EMT effect, reduces tumor proliferation, and obstructs the degradation of the extracellular matrix to prevent metastasis, providing a novel approach to sensitize diverse tumor types to conventional anticancer agents.

#### Small Interfering RNA–Based Suppression of Redundant Oncogenic Signaling Networks: Impairing Parallel Power Supply

Oncogenes and tumor suppressor disruptions collectively establish resilient signaling architectures that enable acquired chemoresistance by ensuring continuous survival and proliferation signaling. The 2 dominant molecular engines of this process are the PI3K/Akt/mTOR survival pathway and the MAPK/ERK proliferation cascade, each maintained in a constitutively active state through distinct genetic mechanisms.^37^ PI3K signaling becomes permanently activated either via PIK3CA oncogenic mutations or through the loss of PTEN, which removes the critical inhibitory checkpoint and results in persistent Akt and mTOR activation.^37^ MAPK pathway hyperactivation similarly arises from activating mutations in KRAS, NRAS, and HRAS, driving ligand-independent ERK stimulation.^38^ Importantly, adaptive RTK reactivation (EGFR, HER2, and MET) further boosts these cascades following targeted therapy, enabling bypass signaling even when upstream inhibition is therapeutically achieved.^39^ Master oncogenic regulators enforce this resistance state: MYC amplifies metabolic rate, chromatin accessibility, and proliferation, while NF-κB sustains anti-apoptotic transcriptional responses and inflammatory cytokine circuits that maintain tumor fitness under drug pressure.^40^ Through this parallel, redundant system of constitutive signaling and transcriptional enforcement, tumors effectively circumvent single-agent blockade and maintain viability against diverse anticancer interventions.

To dismantle the “parallel power supply” of oncogenic signaling, siRNA-mediated intervention is used to simultaneously silence redundant survival cascades and master transcriptional regulators. By degrading the mRNA of key kinases such as PI3K, Akt, or mTOR, siRNA deactivates the metabolic and anti-apoptotic signals that allow tumors to survive therapeutic stress. This approach is especially attractive in preclinical models where phosphatase and tensin homolog (PTEN) loss or PIK3CA mutations establish a state of permanent activation that conventional small-molecule inhibitors often fail to suppress completely.^41^

Furthermore, siRNA is uniquely suited to address the challenge of adaptive resistance and bypass signaling. By targeting the mRNA of RAS isoforms (KRAS, NRAS) and downstream ERK, siRNA can shut down ligand-independent proliferation that often follows the blockade of upstream receptors like EGFR or HER2. For instance, Strand et al utilized peptide-based nanoparticles to deliver KRAS siRNA to pancreatic adenocarcinoma cells, resulting in significantly reduced KRAS expression and cell viability.^42^ Recent evidence also underscores the potential of dual-targeting; Chareddy et al employed chimeric RNAi molecules to simultaneously silence MYC and KRAS across multiple cancer models, successfully halting tumor progression by collapsing these interconnected signaling hubs.^43^

Beyond kinase cascades, siRNA is being deployed to neutralize master oncogenic regulators like MYC, which amplifies metabolic rates and proliferation. Silencing MYC in platinum-resistant ovarian cancer and acute myeloid leukemia has been shown to promote apoptosis and inhibit cell growth, effectively stripping the tumor of its transcriptional enforcement.^44^ By concurrently targeting core signaling pathways and dominant transcriptional regulators, siRNA-based strategies have demonstrated the capacity to destabilize oncogenic resistance networks in preclinical models, thereby resensitizing tumors to standard therapies.

### Small Interfering RNA–Based Clinical Strategies to Overcome Therapeutic Resistance in Cancer

Recent clinical investigations highlight a focused yet emerging application of siRNA therapeutics in addressing key mechanisms of therapeutic resistance (Table 1). Since 2021, siRNA-based clinical trials in oncology have primarily concentrated on targets that are difficult to modulate using conventional pharmacological approaches, including oncogenic drivers, immune checkpoint regulators, and components of transcriptional and immune regulatory networks.

Several studies aim to suppress persistent oncogenic signaling that underlies resistance to targeted and cytotoxic therapies. Notably, exosome-mediated delivery of KRASG12D-specific siRNA is being evaluated in metastatic pancreatic cancer, a setting in which constitutive KRAS activation confers resistance to multiple lines of therapy.^45^ This approach aims to directly reduce oncogene expression, rather than inhibiting downstream effectors, thereby addressing a long-standing limitation of small-molecule inhibitors.

A parallel strategy focuses on immune-mediated resistance. siRNA targeting PD-1 in tumor-infiltrating lymphocytes represents a novel means of modulating immune exhaustion at the cellular level, potentially complementing or extending the activity of antibody-based immune checkpoint inhibitors. Similarly, ex vivo silencing of the E3 ubiquitin ligase Cbl-b in autologous immune cells is under investigation as a method to enhance antitumor immune responses and counteract tumor-induced immunosuppression.

Beyond signaling and immune regulation, siRNA approaches are also being applied to transcriptional mechanisms associated with resistance. Targeting NUDT21, a key regulator of alternative polyadenylation, addresses a non-genetic mode of transcriptional plasticity that enables tumor cells to evade therapeutic control. In addition, bifunctional oligonucleotide constructs incorporating STAT3-directed siRNA have entered early-phase evaluation in refractory lymphomas, reflecting efforts to disrupt survival pathways that contribute to resistance to radiotherapy and immunotherapy.

Collectively, these trials underscore the potential of siRNA-based therapeutics to interrogate and modulate resistance mechanisms that are inaccessible or incompletely addressed by existing drugs. While these studies remain largely early phase, they provide proof-of-concept evidence that gene-silencing strategies can be deployed clinically to target adaptive, immune, and transcriptional drivers of treatment resistance.

### Approved Small Interfering RNA Drugs: Lessons for Oncology

As of late 2025, 6 siRNA therapeutics have gained FDA approval: Patisiran, Givosiran, Lumasiran, Inclisiran, Vutrisiran, and Nedosiran, all targeting hepatic mRNAs for metabolic or genetic disorders such as transthyretin amyloidosis, hypercholesterolemia, and primary hyperoxaluria.^46^ While none are indicated for cancer or resistance mechanisms, their regulatory success validates RNA interference as a clinically viable modality capable of sustained, sequence-specific mRNA knockdown in humans.

The progression from lipid nanoparticle delivery (e.g., Patisiran) to ligand-conjugated siRNAs (e.g., GalNAc-siRNA such as Inclisiran and Nedosiran) demonstrates how targeted chemistry can enhance stability, cellular uptake, and pharmacodynamic durability.^46^These advances underscore the potential of siRNA platforms to achieve durable suppression of gene expression with relatively infrequent dosing, a feature that could be leveraged in oncology to target drivers of resistance.

Importantly, ongoing research efforts are now exploring delivery strategies beyond the liver, such as exosome-based vehicles and tumor-targeted nanoparticles, to reach extrahepatic tissues and resistant tumor cells. Although these strategies are in early clinical stages, they build on the safety and delivery principles established by approved siRNA therapeutics and aim to extend RNAi to complex oncology targets that contribute to bypass signaling, immune escape, and multidrug resistance.

### Small Interfering RNA Therapeutics in Clinical Development

**Figure 2.**
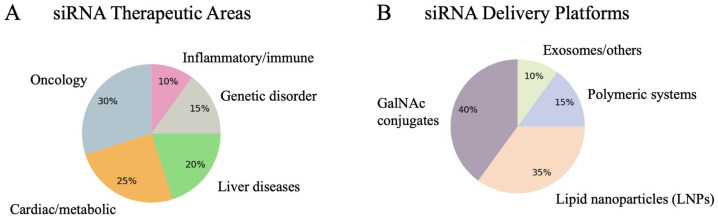
Landscape of siRNA Therapeutic Applications and Delivery Platforms in Clinical Development. (A) Therapeutic areas targeted by siRNA-based interventions. (B) Delivery platforms employed in clinical siRNA development.

Analysis of ongoing and completed clinical trials reveals that oncology, cardiometabolic disorders, and liver diseases represent the most actively pursued therapeutic areas, reflecting both unmet clinical need and favorable target accessibility. Parallel advancements in delivery technologies, particularly GalNAc conjugates and lipid nanoparticles, have substantially improved tissue specificity, pharmacokinetics, and safety profiles, enabling systemic administration and durable gene silencing. Emerging platforms, including polymer-based carriers and extracellular vesicles, further expand the versatility of siRNA therapeutics. Collectively, these developments underscore the growing clinical maturity of RNA interference strategies and highlight their increasing role in precision medicine for both genetic and acquired diseases (Figure 2). Clinical trial data covered all drugs in clinical trials from 2020-2025 and can be found in Supplementary Table 1.

### Challenges and Future Perspectives for Oncology Small Interfering RNA Therapeutics

Despite the clinical success of hepatic siRNA therapies, extending these results to oncology faces significant translational bottlenecks. Systemic administration of siRNA to extrahepatic tissues is limited by rapid renal clearance, nuclease-mediated degradation, and unintended sequestration by the liver or immune system. Even when siRNA reaches the tumor site, it must overcome the “last mile” barrier of endosomal escape to reach the cytosol and engage the RISC. Nanotechnology-enhanced delivery strategies, including exosome-mimicking systems, cell-membrane-coated nanoparticles, and ligand-targeted formulations, are being actively explored to improve circulation half-life, enhance tumor targeting, and facilitate cytosolic release.^47^

Artificial intelligence (AI) and machine learning (ML) emerge as critical tools to accelerate the development of next-generation siRNA therapeutics.^48^ ML models are applied to predict siRNA potency and minimize off-target effects, enabling efficient selection of highly active sequences.^48^ In parallel, AI-driven computational frameworks are used to optimize nanoparticle formulations, including lipid composition, surface chemistry, particle size, and release kinetics to maximize cellular uptake and tumor penetration while minimizing toxicity.^49^ By integrating sequence design with rational delivery engineering, these AI-enabled strategies are shifting siRNA development from empirical trial-and-error toward predictive, data-driven approaches.

## Conclusion

Therapeutic resistance remains a central obstacle in oncology, driven by complex and adaptive molecular networks that enable tumor survival under sustained treatment pressure. Conventional pharmacological approaches, while effective initially, are frequently undermined by pathway redundancy, transcriptional plasticity, and the functional inaccessibility of key resistance drivers. In this context, siRNA-based therapeutics offer a fundamentally different strategy by directly suppressing resistance-associated gene expression at the mRNA level, including targets that are traditionally considered undruggable.

As outlined in this review, siRNA interventions have demonstrated the capacity to disrupt multiple resistance mechanisms, ranging from drug efflux and apoptotic evasion to DNA damage repair, epigenetic remodeling, epithelial-mesenchymal transition, and constitutive oncogenic signaling. Preclinical studies consistently show that silencing these pathways can restore sensitivity to chemotherapy and targeted therapy. Emerging clinical trials further support the feasibility of siRNA approaches in oncology, particularly for targeting oncogenic drivers, immune regulatory checkpoints, and transcriptional regulators that escape conventional inhibition.

Several significant challenges remain, including efficient extrahepatic delivery, tumor-specific targeting, and endosomal escape; however, ongoing advances in nanotechnology and ligand-directed delivery systems are rapidly addressing these limitations. Collectively, siRNA-based strategies hold substantial promise for reshaping resistance management in cancer and may ultimately enable more durable and personalized therapeutic responses when integrated into future oncology treatment paradigms.

## Supplementary Materials

Supplementary Material

## Figures and Tables

**Table 1.** Representative Clinical Trials (Since 2021) Employing siRNA-Based Strategies to Address Therapeutic Resistance Mechanisms in Cancer and Immune-Mediated Disease

**Phase**	**NCT Number**	**Target / siRNA Strategy**	**Indication**	**Resistance Mechanism Addressed**
I/II	NCT03608631	KRASG12D siRNA (iExosomes)	Metastatic pancreatic cancer	Addresses persistent oncogenic signaling driven by KRAS mutations that are refractory to small-molecule inhibition. Evaluates exosome-mediated delivery of siRNA to suppress KRAS expression in patients with disease progression following prior systemic therapies.
I	NCT05902520	PD-1 siRNA (PH-762, INTASYL)	Advanced solid tumors	Investigates siRNA-mediated silencing of PD-1 in tumor-infiltrating lymphocytes as an approach to modulate immune exhaustion and adaptive resistance to immune checkpoint blockade.
I	NCT06424301	NUDT21 siRNA	Resistant retinoblastoma	Targets alternative polyadenylation machinery implicated in transcriptional plasticity and treatment resistance. Explores whether modulation of mRNA processing can restore therapeutic sensitivity in refractory pediatric ocular tumors.
I	NCT04995536	STAT3 siRNA–CpG conjugate	Refractory lymphomas	Evaluates a bifunctional oligonucleotide combining STAT3-targeting siRNA with immune-stimulatory CpG motifs to suppress STAT3-dependent survival pathways associated with resistance to radiotherapy and immunotherapy.
I	NCT06172894	Cbl-b siRNA (APN401, ex vivo)	Advanced solid tumors	Phase Ib trial of autologous PBMCs transfected ex vivo with Cbl-b siRNA to enhance T-cell antitumor activity. Preliminary reports indicate manageable safety and early signals of disease stabilization in a subset of heavily pre-treated patients, accompanied by increases in cytotoxic T-cell biomarkers.

## Data Availability

The data that support the findings of this study are available on request from the corresponding author.
