# Supplementary material for: Small Interfering RNA Strategies to Overcome Drug Resistance in Cancer: Pathway Targeting and Translational Advances
Source: Eurasian J Med. 2026 Feb 18;58(1):e251350. doi: 10.5152/eurasianjmed.2026.251350 (PMC13187683; doi:10.5152/eurasianjmed.2026.251350)
Supplement: Supplementary Material [file supplementary_material.pdf]

Supplementary Table 1. SiRNA Clinical Trials (2020-2025)

Clinical Trial ID	Description	Reference
**NCT06154278**<br>Sponsor: University of Maryland, Baltimore<br>Recruitment Status: Recruiting<br>Phase: II<br>Start/Completion Date:<br>July 25th 2024 to<br>July 1st 2027	This trial evaluates intrahepatic and peripheral responses to siRNA, Imdusiran (AB-729), in patients with chronic Hepatitis B.	[1]
**NCT06424301**<br>Sponsor: Eye & ENT Hospital of Fudan University<br>Recruitment Status: Recruiting<br>Phase: I<br>Start/Completion Date:<br>December 20th 2024 to September 30th 2025	This trial evaluates the short-term safety and preparatory potency of NUDT21 siRNA in patients with resistant retinoblastoma, and to confirm future large-scale clinical trials.	[2]
**NCT05844293**<br>Sponsor: Sirnaomics<br>Recruitment Status: Recruiting<br>Phase: I<br>Start/Completion Date:<br>May 16th 2023 to June 2024	This study assesses the pharmacokinetics, pharmacodynamics, safety, and tolerability of single ascending dose (SAD) of FXI-GalNAc-siRNA when administered subcutaneously to healthy people.	[3]
**NCT05438069**<br>Sponsor: Jena University Hospital<br>Recruitment Status: Recruiting<br>Phase: N/A (Not Applicable)<br>Start/Completion Date:<br>December 11th 2020 to December 11th 2025	This study evaluates the hypercholesterinemia following to the administration of siRNA Inclisiran in German patients.	[4]
**NCT06138964**<br>Sponsor: National Skin Centre<br>Recruitment Status: Recruiting<br>Phase: II<br>Start/Completion Date:<br>November 14th 2023 to November 14th 2024	This study compares the effect of siRNA against the secreted protein acidic and cysteine rich SPARC coding gene (siSPARC) microneedle patch versus siRNA against SPARC and interleukin-4 receptor alpha (IL4-RA) (siSPARC + siLR4A) microneedle patch on post-surgical scars formation.	[5]
**NCT06586684**<br>Sponsor: First Affiliated Hospital of Xinjiang Medical University<br>Recruitment Status: Not yet recruiting<br>Phase: IV<br>Start/Completion Date:<br>September 20th 2024 to September 30th 2026	This study assesses the effect of siRNA Inclisiran on carotid plaques in patients with atherosclerosis using carotid ultrasound.	[6]
**NCT03608631**<br>Sponsor: M.D. Anderson Cancer Center<br>Recruitment Status: Recruiting<br>Phase: I/II<br>Start/Completion Date:<br>January 27th 2021 to April 30th 2027	This study evaluates the effect of dose-escalation of mesenchymal stromal cells-derived exosomes with KRASG12D siRNA for patients with metastatic pancreas cancer enclosing KRASG12D mutation.	[7]
**NCT06960213**<br>Sponsor: ADARx Pharmaceuticals, Inc.<br>Recruitment Status: Recruiting<br>Phase: III<br>Start/Completion Date:<br>August 28th 2025 to December 31st 2026	The STOP-HAE study evaluates the efficacy of siRNA (ADX-324) targeting the coagulating factor (Prekallikrein) in Hereditary Angioedema participants. The study also assesses PK, PD, safety, and patient-reported outcome measures.	[8]

Clinical Trial ID	Description	Reference
**NCT05519475**<br>Sponsor: Regeneron Pharmaceuticals<br>Recruitment Status: Recruiting<br>Phase: II<br>Start/Completion Date:<br>February 9th 2023 to September 8th 2027	This study evaluates siRNA for the treatment of metabolic dysfunction-associated steatohepatitis (MASH) in patients with genetic predisposition factors.	[9]
**NCT06923280**<br>Sponsor: Huashan Hospital<br>Recruitment Status: Not yet recruiting<br>Phase: N/A<br>Start/Completion Date:<br>May 1st 2025 to May 31st 2028	This study compares successive pegylated interferon (PEG-IFNα) therapy protocols in chronic hepatitis B (CHB) patients formerly treated with antisense oligonucleotide (ASO) or siRNA.	[10]
**NCT05902520**<br>Sponsor: AgonOx, Inc.<br>Recruitment Status: Recruiting<br>Phase: I<br>Start/Completion Date:<br>June 19th 2023<br>May 19th 2026	This study evaluates the adoptive transfer of immune markers (CD39, CD103, CD8) along with cancer-isolated T cells (AGX148) alone or in combination with siRNA modulation of a novel cancer immunotherapy approach PD-1 (AGX148/PH-762) combining a tumor-specific Tumor-Infiltrating Lymphocyte (TIL) product (AGX148) and siRNA targeting the PD-1 immune checkpoint on those T-cells (PH-762) in patients with advanced solid cancers.	[11]
**NCT05648214**<br>Sponsor: Regeneron Pharmaceuticals<br>Recruitment Status: Active, not recruiting<br>Phase: I<br>Start/Completion Date:<br>December 27th 2022 to October 18th 2025	This study evaluates the PK, safety, and tolerability of ALN-PNP, a siRNA targeting patatin-like phospholipase domain-containing protein-3 (PNPLA3), in healthy subjects with non-alcoholic fatty liver disease (NAFLD).	[12]
**NCT07187401**<br>Sponsor: Regeneron Pharmaceuticals<br>Recruitment Status: Recruiting<br>Phase: I/II<br>Start/Completion Date:<br>November 28th 2025 to January 4th 2030	This study evaluates the PK, safety, tolerability, and efficacy of a siRNA treatment against complement factor B (ALN-CFB) in patients with paroxysmal nocturnal hemoglobinuria and persistent anemia with C5-inhibitor therapy	[13]
**NCT06024408**<br>Sponsor: Regeneron Pharmaceuticals<br>Recruitment Status: Completed<br>Phase: I<br>Start/Completion Date:<br>May 21st 2024 to<br>July 7th 2025	This study evaluates the PK, PD, safety, and tolerability of ALN-PNP siRNA in subjects NAFLD and a PNPLA3 genetic risk factor	[14]
**NCT04601844**<br>Sponsor: Regeneron Pharmaceuticals<br>Recruitment Status: Completed<br>Phase: I<br>Start/Completion Date:<br>November 16th 2020 to July 23rd 2021	This study evaluates the PK, PD, safety, and tolerability of single doses of subcutaneously administered human monoclonal antibody (Pozelimab) combined with single doses of subcutaneously administered siRNA (Cemdisiran) in healthy subjects	[15]
**NCT05876312**<br>Sponsor: ADARx Pharmaceuticals, Inc.<br>Recruitment Status: Recruiting<br>Phase: I/II<br>Start/Completion Date:<br>August 7th 2023 to<br>September 30th 2026	This study evaluates the PK, PD, safety, and tolerability of siRNA (ADX-038) in both healthy participants and in in patients with paroxysmal nocturnal hemoglobinuria (PNH).	[16]

Clinical Trial ID	Description	Reference
**NCT05637255**<br>Sponsor: Sylentis, S.A.<br>Recruitment Status: Recruiting<br>Phase: II<br>Start/Completion Date:<br>November 22nd 2022 to<br>July 22nd 2023	This study evaluates the safety and efficacy of siRNA (SYL1801) eye drops doses in patients with neovascular age-related macular degeneration (AMD).	[17]
**NCT07271186**<br>Sponsor: Regeneron Pharmaceuticals<br>Recruitment Status: Not yet recruiting<br>Phase: II<br>Start/Completion Date:<br>2025-12-09<br>2027-12-08<br>December 9th 2025 to December 8th 2027	This study evaluates the safety, efficacy, PK, and PD effects of ANGPTL3 inhibition with siRNA alone or in combination with an ANGPTL3 antibody in patients with diabetic kidney disease.	[18]
**NCT06845202**<br>Sponsor: Alnylam Pharmaceuticals<br>Recruitment Status: Recruiting<br>Phase: I/II<br>Start/Completion Date:<br>March 3rd 2025 to August 31st 2027	This study evaluates the efficacy, safety, tolerability, PK, and PD of a single dose of siRNA (ALN-4324) in overweight and obese healthy subjects and it evaluates the multiple doses of ALN-4324 in overweight and obese patients with type 2 diabetes mellitus (T2DM)	[19]
**NCT05661916**<br>Sponsor: Alnylam Pharmaceuticals<br>Recruitment Status: Completed<br>Phase: I<br>Start/Completion Date:<br>January 16th 2023 to September 30th 2025	This study evaluates the safety, tolerability, PK, and PD of a single dose of subcutaneously administration of siRNA (ALN-TTRSC04) in healthy participants.	[20]
**NCT05783206**<br>Sponsor: National Research Center - Institute of Immunology Federal Medical-Biological Agency of Russia<br>Recruitment Status: Completed<br>Phase: II/III<br>Start/Completion Date:<br>February 10th 2022 to<br>July 28th 2023	This study evaluates the safety and efficacy of aerosolized siRNA (MIR 19®) inhalation in mild COVID-19 patients.	[21]
**NCT05256810**<br>Sponsor: Alnylam Pharmaceuticals<br>Recruitment Status: Terminated<br>Phase: I/II<br>Start/Completion Date:<br>February 25th 2022 to<br>January 25th 2023	This study evaluates the efficacy, safety, tolerability, PK, and PD of a single escalating dose and multiple doses of siRNA (ALN-XDH) in healthy participants and gout patients.	[22]
**NCT05208996**<br>Sponsor: National Research Center - Institute of Immunology Federal Medical-Biological Agency of Russia<br>Recruitment Status: Completed<br>Phase: I<br>Start/Completion Date:<br>January 22nd 2021 to<br>March 26th 2021	This study evaluates the safety of administration of siRNA of SARS-CoV-2 (siCoV) uploaded on cationic peptide dendrimer (KK-46) to determine the highest daily dose as a single therapy in healthy subjects.	[23]

Clinical Trial ID	Description	Reference
**NCT05184127**<br>Sponsor: National Research Center - Institute of Immunology Federal Medical-Biological Agency of Russia<br>Recruitment Status: Completed<br>Phase: II<br>Start/Completion Date:<br>April 27th 2021 to September 7th 2021	This study evaluates the safety and efficacy of aerosolized siRNA (MIR 19®) inhalation in moderate COVID-19 patients who didn't require admission to the intensive care unit.	[24]
**NCT04765657**<br>Sponsor: Novartis Pharmaceuticals<br>Recruitment Status: Active, not recruiting<br>Phase: III<br>Start/Completion Date:<br>March 1st 2021<br>December 28th 2026	This study evaluates the efficacy and safety of siRNA (Inclisiran) in Asian patients with atherosclerotic cardiovascular disease (ASCVD) or in ASCVD high risk and elevated low-density lipoprotein cholesterol patients as complementary to diet accompanied with maximum dose of statins with or without adjunct lipid-lowering agent (ORION-18).	[25]
**NCT05974345**<br>Sponsor: Novartis Pharmaceuticals<br>Recruitment Status: Completed<br>Phase: Observational<br>Start/Completion Date:<br>November 3rd 2023 to December 15th 2023	This virtual study predicts the efficacy of siRNA (Inclisiran) on serious adverse CV effects and CV death in virtual patients with ASCVD and elevated LDL-C.	[26]
**NCT05888103**<br>Sponsor: Novartis Pharmaceuticals<br>Recruitment Status: Completed<br>Phase: III<br>Start/Completion Date:<br>July 11th 2023 to October 24th 2024	This study evaluates the safety and efficacy of siRNA (inclisiran) as a single treatment in Chinese patients with low or moderate ASCVD risk and elevated LDL-C and lacking any lipid lowering treatment.	[27]
**NCT05310422**<br>Sponsor: Sylentis, S.A.<br>Recruitment Status: Completed<br>Phase: III<br>Start/Completion Date:<br>March 24th 2022 to October 11th 2023	This study evaluates the safety of siRNA (tivanisiran sodium) eye drops administered once daily for 24 months in dry eye disease (DED) participants.	[28]
**NCT04877756**<br>Sponsor: Olix Pharmaceuticals, Inc.<br>Recruitment Status: Completed<br>Phase: II<br>Start/Completion Date:<br>August 19th 2021<br>July 7th 2023	This study evaluates the efficacy of siRNA (OLX10010) as an additional treatment to prevent the recurrence of hypertrophic scars following revision surgery	[29]
**NCT04819269**<br>Sponsor: Sylentis, S.A.<br>Recruitment Status: Completed<br>Phase: III<br>Start/Completion Date:<br>May 25th 2021 to December 11th 2023	This study evaluates the efficacy and safety of siRNA (tivanisiran sodium) eye drops after 2-week administered once daily for 3 months in DED subjects due to Sjögren's Syndrome.	[30]

Clinical Trial ID	Description	Reference
**NCT05761301**<br>Sponsor: Alnylam Pharmaceuticals<br>Recruitment Status: Terminated<br>Phase: I/II<br>Start/Completion Date:<br>March 10th 2023 to<br>April 3rd 2025	This study evaluates the efficacy, safety, tolerability, PK, and PD of a single dose of siRNA (ALN- KHK) in overweight and obese healthy subjects and it evaluates the multiple doses of ALN- KHK in overweight and obese patients with type 2 diabetes mellitus (T2DM)	[31]
**NCT04666298**<br>Sponsor: Novartis Pharmaceuticals<br>Recruitment Status: Completed<br>Phase: II<br>Start/Completion Date:<br>January 29th 2021 to<br>October 19th 2022	This study evaluates the effect of different doses of siRNA (Inclisiran) administered subcutaneously in Japanese subjects with high cardiovascular (CV) risk and elevated Low-density lipoprotein cholesterol (LDL-C)	[32]
**NCT06423352**<br>Sponsor: Alnylam Pharmaceuticals<br>Recruitment Status: Completed<br>Phase: I/II<br>Start/Completion Date:<br>June 5th 2024 to<br>July 17th 2025	This study evaluates the efficacy, safety, tolerability, PK, and PD of siRNA (Zilebesiran) in Japanese patients with mild to moderate hypertension	[33]
**NCT05484206**<br>Sponsor: Vir Biotechnology, Inc.<br>Recruitment Status: Recruiting<br>Phase: I<br>Start/Completion Date:<br>September 21st 2022 to<br>April 30th 2027	This study evaluates the safety and PK of siRNA (VIR-2218) and (VIR-3434) as a single therapy and adjunct Therapy in hepatic dysfunction patients	[34]
**NCT05103332**<br>Sponsor: Alnylam Pharmaceuticals<br>Recruitment Status: Completed<br>Phase: II<br>Start/Completion Date:<br>November 5th 2021 to September 13th 2024	This study evaluates the efficacy and safety of siRNA (Zilebesiran) as an adjunct treatment in hypertension patients who aren't properly treated by antihypertensive medication	[35]
**NCT04936035**<br>Sponsor: Alnylam Pharmaceuticals<br>Recruitment Status: Completed<br>Phase: II<br>Start/Completion Date:<br>July 7th 2021 to<br>December 5th 2024	This study evaluates the efficacy and safety of siRNA (ALN-AGT01) in patients with mild to moderate hypertension	[36]
**NCT06115967**<br>Sponsor: AstraZeneca<br>Recruitment Status: Completed<br>Phase: I<br>Start/Completion Date:<br>November 15th 2023 to<br>October 16th 2025	This study evaluates the safety, tolerability, PK, and PD of an escalating dose of subcutaneous siRNA (AZD6912) in healthy subjects.	[37]

Clinical Trial ID	Description	Reference
**NCT04152200**<br>Sponsor: Alnylam Pharmaceuticals<br>Recruitment Status: Completed<br>Phase: III<br>Start/Completion Date:<br>January 21st 2020 to<br>June 23rd 2025	This study evaluates the efficacy, safety, PK, and PD of siRNA (Lumasiran) in advanced primary hyperoxaluria type 1 (PH1) patients.	[38]
**NCT04659863**<br>Sponsor: Novartis Pharmaceuticals<br>Recruitment Status: Completed<br>Phase: III<br>Start/Completion Date:<br>February 16th 2021 to<br>November 18th 2024	This study evaluates the efficacy, safety, and tolerability of siRNA (Inclisiran) in homozygous familial hypercholesterolemia (HoFH) and elevated low density lipoprotein cholesterol (LDL-C) adolescents.	[39]
**NCT04652726**<br>Sponsor: Novartis Pharmaceuticals<br>Recruitment Status: Completed<br>Phase: III<br>Start/Completion Date:<br>January 27th 2021 to<br>November 27th 2024	This study evaluates the efficacy, safety, and tolerability of siRNA (Inclisiran) in heterozygous familial hypercholesterolemia (HeFH) and elevated low density lipoprotein cholesterol (LDL-C) adolescents.	[40]
**NCT06597019**<br>Sponsor: Novartis Pharmaceuticals<br>Recruitment Status: Recruiting<br>Phase: III<br>Start/Completion Date:<br>December 9th 2024 to April 15th 2029	This study evaluates the efficacy, tolerability, and safety of siRNA (Inclisiran) in children (aged 6 -12 years) with HeFH and elevated LDL-C.	[41]
**NCT06597006**<br>Sponsor: Novartis Pharmaceuticals<br>Recruitment Status: Recruiting<br>Phase: III<br>Start/Completion Date:<br>2025-02-28<br>2029-04-15<br>February 28th 2025 to<br>April 15th 2029	This study evaluates the efficacy, tolerability, and safety of siRNA (Inclisiran) in children (aged 6 -12 years) with HoFH and elevated LDL-C.	[42]
**NCT06452771**<br>Sponsor: Regeneron Pharmaceuticals<br>Recruitment Status: Recruiting<br>Phase: I<br>Start/Completion Date:<br>June 27th 2024 to<br>December 3rd 2026	This study evaluates the Pk, safety, and tolerability of siRNA (ALN-ANG3) in healthy subjects.	[43]
**NCT07181109**<br>Sponsor: Alnylam Pharmaceuticals<br>Recruitment Status: Recruiting<br>Phase: III<br>Start/Completion Date:<br>September 22nd 2025 to<br>October 29th 2030	This study evaluates the safety and efficacy of siRNA (Zilebesiran) in decreasing serious adverse CV events in adult subjects with uncontrolled hypertension accompanied with diagnosed cardiovascular disease (CVD) or risky CVD.	[44]

Clinical Trial ID	Description	Reference
**NCT05030428**<br>Sponsor: Novartis Pharmaceuticals<br>Recruitment Status: Active, not recruiting<br>Phase: III<br>Start/Completion Date:<br>November 23rd 2021 to<br>October 13th 2027	This study evaluates the effect of siRNA (Inclisiran) on serious CV events in diagnosed (CVD) patients.	[45]
**NCT04782271**<br>Sponsor: Sylentis, S.A.<br>Recruitment Status: Completed<br>Phase: I<br>Start/Completion Date:<br>March 17th 2021 to<br>December 21st 2021	This study evaluates the tolerability, safety, and PK of doses of siRNA (SYL1801) eye drops in healthy subjects.	[46]
**NCT06272487**<br>Sponsor: Alnylam Pharmaceuticals<br>Recruitment Status: Active, not recruiting<br>Phase: II<br>Start/Completion Date:<br>February 29th 2024 to<br>December 19th 2025	This study evaluates the effect of siRNA (Zilebesiran) as an additional therapy in patients with CV risk and hypertension not properly regulated by antihypertensive medications.	[47]
**NCT05643118**<br>Sponsor: Olix Pharmaceuticals, Inc.<br>Recruitment Status: Completed<br>Phase: I<br>Start/Completion Date:<br>January 4th 2023 to<br>November 10th 2025	This study evaluates the tolerability and safety of siRNA (OLX10212) in AMD patients.	[48]
**NCT06585449**<br>Sponsor: Alnylam Pharmaceuticals<br>Recruitment Status: Recruiting<br>Phase: I<br>Start/Completion Date:<br>October 14th 2024 to July 5th 2028	This study evaluates the tolerability, safety, PD, and PK of a single dose of siRNA (ALN-HTT02) in Huntington's disease patients.	[49]
**NCT06172894**<br>Sponsor: invIOs GmbH<br>Recruitment Status: Completed<br>Phase: I<br>Start/Completion Date:<br>August 22nd 2023<br>October 1st 2024	This study evaluates the dose of siRNA (APN401) in advanced solid cancer patients.	[50]
**NCT04270760**<br>Sponsor: Amgen<br>Recruitment Status: Completed<br>Phase: II<br>Start/Completion Date:<br>July 28th 2020 to November 8th 2022	This study evaluates the efficacy, tolerability, and safety of siRNA (Olpasiran) (AMG 890) in high Lipoprotein patients.	[51]

Clinical Trial ID	Description	Reference
**NCT04555486**<br>Sponsor: Dicerna Pharmaceuticals, Inc., a Novo Nordisk company<br>Recruitment Status: Completed<br>Phase: I<br>Start/Completion Date:<br>September 14th 2020 to September 7th 2021	This study evaluates the tolerability, safety, PK, and PD of a single dose of siRNA (DCR-PHXC) in primary hyperoxaluria type 3 patients.	[52]

References

1. ClinicalTrials.gov. Intrahepatic and Peripheral Responses to Imdusiran (AB-729) in Chronic Hepatitis B (i-LIVER); available from: https://clinicaltrials.gov/study/NCT06154278 (accessed on 12 December 2025).

2. ClinicalTrials.gov. Targeting NUDT21 siRNA Drugs for Patients With Refractory Retinoblastoma; available from: https://clinicaltrials.gov/study/NCT06424301 (accessed on 12 December 2025).

3. ClinicalTrials.gov. Single Ascending Dose of FXI-GalNAc-siRNA in Healthy Subjects; available from: https://clinicaltrials.gov/study/NCT05844293 (accessed on 12 December 2025).

4. ClinicalTrials.gov. German Inclisiran Network: Retrospective Registry of Patients Being Treated With the siRNA Inclisiran in Germany (GIN); available from: https://clinicaltrials.gov/study/NCT05438069 (accessed on 13 December 2025).

5. ClinicalTrials.gov. Comparing the Effect of siSPARC Microneedle Patch Versus siSPARC+siLR4A Microneedle Patch on Post-surgical Scars; available from: https://www.clinicaltrials.gov/study/NCT06138964 (accessed on 13 December 2025).

6. ClinicalTrials.gov. Effect of Small Interfering RNA Inclisiran on Carotid Plaques As Assessed by Carotid Ultrasound; available from: https://clinicaltrials.gov/study/NCT06586684 (accessed on 13 December 2025).

7. ClinicalTrials.gov. iExosomes in Treating Participants With Metastatic Pancreas Cancer With KrasG12D Mutation; available from: https://www.clinicaltrials.gov/study/NCT03608631 (accessed on 13 December 2025).

8. ClinicalTrials.gov. STOP-HAE: A Phase 3 Study of ADX-324 in HAE (STOP-HAE); available from: https://clinicaltrials.gov/study/NCT06960213 (accessed on 13 December 2025).

9. ClinicalTrials.gov. A Precision Medicine Approach Using Gene Silencing to Treat a Chronic Liver Disease Called Metabolic Dysfunction-Associated Steatohepatitis (MASH) in Adult Participants at Increased Genetic Risk for This Condition (NASHGEN-2); available from: https://clinicaltrials.gov/study/NCT05519475 (accessed on 13 December 2025).

10. ClinicalTrials.gov. Sequential PEG-IFN for HBV After Ending RNA-targeted Regimens; available from: https://clinicaltrials.gov/study/NCT06923280 (accessed on 13 December 2025).

11. ClinicalTrials.gov. Adoptive Cell Therapy Using Cancer Specific CD8+ Tumor Infiltrating Lymphocytes in Adult Patients With Solid Tumors (ACT); available from: https://clinicaltrials.gov/study/NCT05902520 (accessed on 13 December 2025).

12. ClinicalTrials.gov. A Trial to Learn if ALN-PNP is Safe and Well Tolerated in Healthy Adults and Adult Participants With Non-Alcoholic Fatty Liver Disease (NAFLD); available from: https://clinicaltrials.gov/study/NCT05648214 (accessed on 13 December 2025).

13. ClinicalTrials.gov. A First-in-Human Safety and Efficacy Study of ALN-CFB, a Small Interfering RNA (siRNA) Targeting Complement Factor B, in Adult Participants With Paroxysmal Nocturnal Hemoglobinuria With Persistent Anemia on a C5 Inhibitor; available from: https://clinicaltrials.gov/study/NCT07187401 (accessed on 13 December 2025).

14. ClinicalTrials.gov. A Trial to Learn if Receiving ALN-PNP siRNA is Safe and Well Tolerated, and How it Works in Adult Participants With Nonalcoholic Fatty Liver Disease (NAFLD) and a Genetic Risk Factor; available from: https://clinicaltrials.gov/study/NCT06024408 (accessed on 13 December 2025).

15. ClinicalTrials.gov. Safety, Tolerability, Pharmacokinetics, and Pharmacodynamics of Pozelimab in Combination With Cemdisiran in Healthy Adult Volunteers; available from: https://clinicaltrials.gov/study/NCT04601844 (accessed on 13 December 2025).

16. ClinicalTrials.gov. Safety, Tolerability, PK and PD of ADX-038 in Healthy Participants and Paroxysmal Nocturnal Hemoglobinuria (PNH) Patients; available from: https://clinicaltrials.gov/study/NCT05876312 (accessed on 13 December 2025).

17. ClinicalTrials.gov. A Randomized, Double Masked, Parallel Group, Dose-finding Study to Evaluate SYL1801 in Patients With Neovascular Age-related Macular Degeneration (AMD); available from: https://clinicaltrials.gov/study/NCT05637255 (accessed on 14 December 2025).

18. ClinicalTrials.gov. Study to Assess the Effects of Angiopoietin-like Protein 3 (ANGPTL3) Inhibition in Adult Participants With Diabetic Kidney Disease (ANCHOR-POC); available from: https://clinicaltrials.gov/study/NCT07271186 (accessed on 14 December 2025).

19. ClinicalTrials.gov. A Study to Evaluate ALN-4324 in Overweight to Obese Healthy Volunteers and in Overweight to Obese Patients With T2DM; available from: https://clinicaltrials.gov/study/NCT06845202 (accessed on 15 December 2025).

20. ClinicalTrials.gov. A Study to Evaluate the Safety, Tolerability, Pharmacokinetics and Pharmacodynamics of ALN-TTRSC04 in Healthy Subjects; available from: https://clinicaltrials.gov/study/NCT05661916 (accessed on 15 December 2025).

21. ClinicalTrials.gov. Evaluation of Safety & Efficacy of MIR 19® Inhalation Solution in Patients With Mild COVID-19; available from: https://www.clinicaltrials.gov/study/NCT05783206 (accessed on 15 December 2025).

22. ClinicalTrials.gov. A Study to Evaluate ALN-XDH in Healthy Subjects and Patients With Gout; available from: https://clinicaltrials.gov/study/NCT05256810 (accessed on 15 December 2025).

23. ClinicalTrials.gov. The siCoV/KK46 Drug Open-safety Study; available from: https://clinicaltrials.gov/study/NCT05208996 (accessed on 15 December 2025).

24. ClinicalTrials.gov. Evaluation of Safety & Efficacy of MIR 19® Inhalation Solution in Patients With Moderate COVID-19; available from: https://clinicaltrials.gov/study/NCT05184127 (accessed on 15 December 2025).

25. ClinicalTrials.gov. Study of Efficacy and Safety of Inclisiran in Asian Participants With Atherosclerotic Cardiovascular Disease (ASCVD) or ASCVD High Risk and Elevated Low Density Lipoprotein Cholesterol (LDL-C); available from: https://clinicaltrials.gov/study/NCT04765657 (accessed on 15 December 2025).

26. ClinicalTrials.gov. In Silico Study Assessing the Impact of Inclisiran on Major Adverse Cardiovascular Events in Patients With Established Cardiovascular Disease (SIRIUS); available from: https://clinicaltrials.gov/study/NCT05974345 (accessed on 16 December 2025).

27. ClinicalTrials.gov. Efficacy and Safety of Inclisiran as Monotherapy in Chinese Adults With Low or Moderate ASCVD Risk and Elevated Low-density Lipoprotein Cholesterol (V-Mono China); available from: https://clinicaltrials.gov/study/NCT05888103 (accessed on 16 December 2025).

28. ClinicalTrials.gov. Safety Study of Tivanisiran to Treat Dry Eye (FYDES); available from: https://clinicaltrials.gov/study/NCT05310422 (accessed on 15 December 2025).

29. ClinicalTrials.gov. Study to Evaluate Efficacy of OLX10010 in Reducing Recurrence of Hypertrophic Scarring After Scar Revision Surgery; available from: https://clinicaltrials.gov/study/NCT04877756 (accessed on 15 December 2025).

30. ClinicalTrials.gov. Tivanisiran for Dry Eye in Subjects With Sjögren's Syndrome; available from: https://clinicaltrials.gov/study/NCT04819269 (accessed on 15 December 2025).

31. ClinicalTrials.gov. A Phase 1/2 Study to Evaluate ALN-KHK in Overweight to Obese Healthy Volunteers and Obese Patients With T2DM; available from: https://clinicaltrials.gov/study/NCT05761301 (accessed on 15 December 2025).

32. ClinicalTrials.gov. Study of Efficacy and Safety of Inclisiran in Japanese Participants With High Cardiovascular Risk and Elevated LDL-C (ORION-15); available from: https://clinicaltrials.gov/study/NCT04666298 (accessed on 15 December 2025).

33. ClinicalTrials.gov. A Study to Evaluate Zilebesiran in Japanese Patients With Mild to Moderate Hypertension; available from: https://clinicaltrials.gov/study/NCT06423352 (accessed on 13 December 2025).

34. ClinicalTrials.gov. Effect of Hepatic Impairment on the Pharmacokinetics and Safety of VIR-2218 and VIR-3434; available from: https://www.clinicaltrials.gov/study/NCT05484206 (accessed on 15 December 2025).

35. ClinicalTrials.gov. Zilebesiran as Add-on Therapy in Patients With Hypertension Not Adequately Controlled by a Standard of Care Antihypertensive Medication (KARDIA-2); available from: https://clinicaltrials.gov/study/NCT05103332 (accessed on 15 December 2025).

36. ClinicalTrials.gov. A Study to Evaluate Efficacy and Safety of ALN-AGT01 in Patients With Mild To-Moderate Hypertension (KARDIA-1); available from: https://clinicaltrials.gov/study/NCT04936035 (accessed on 15 December 2025).

37. ClinicalTrials.gov. A Study to Assess the Safety, Tolerability, Pharmacokinetics, Pharmacodynamics of Subcutaneous AZD6912 in Healthy Participants; available from: https://clinicaltrials.gov/study/NCT06115967 (accessed on 16 December 2025).

38. ClinicalTrials.gov. A Study to Evaluate Lumasiran in Patients With Advanced Primary Hyperoxaluria Type 1 (ILLUMINATE-C); available from: https://clinicaltrials.gov/study/NCT04152200 (accessed on 16 December 2025).

39. ClinicalTrials.gov. Study to Evaluate Efficacy and Safety of Inclisiran in Adolescents With Homozygous Familial Hypercholesterolemia (ORION-13); available from: https://clinicaltrials.gov/study/NCT04659863 (accessed on 16 December 2025).

40. ClinicalTrials.gov. Study to Evaluate Efficacy and Safety of Inclisiran in Adolescents With Heterozygous Familial Hypercholesterolemia (ORION-16); available from: https://clinicaltrials.gov/study/NCT04652726 (accessed on 16 December 2025).

41. ClinicalTrials.gov. Study to Evaluate Efficacy and Safety of Inclisiran in Children With Heterozygous Familial Hypercholesterolemia (ORION-20); available from: https://clinicaltrials.gov/study/NCT06597019 (accessed on 16 December 2025).

42. ClinicalTrials.gov. Study to Evaluate Safety, Tolerability and Efficacy of Inclisiran in Children With Homozygous Familial Hypercholesterolemia (ORION-19); available from: https://clinicaltrials.gov/study/NCT06597006 (accessed on 16 December 2025).

43. ClinicalTrials.gov. A First In Human (FIH) Study to Learn if Different Doses of ALN-ANG3 Are Safe and Well Tolerated in Healthy Adults; available from: https://clinicaltrials.gov/study/NCT06452771 (accessed on 16 December 2025).

44. ClinicalTrials.gov. Zilebesiran in Patients With Hypertension Not Adequately Controlled and With Either Established Cardiovascular Disease or High Risk for Cardiovascular Disease (ZENITH); available from: https://clinicaltrials.gov/study/NCT07181109 (accessed on 16 December 2025).

45. ClinicalTrials.gov. Study of Inclisiran to Prevent Cardiovascular (CV) Events in Participants With Established Cardiovascular Disease (VICTORION-2P); available from: https://www.clinicaltrials.gov/study/NCT05030428 (accessed on 16 December 2025).

46. ClinicalTrials.gov. Safety, Tolerability and Pharmacokinetic Profile of SYL1801 Eye Drops; available from: https://clinicaltrials.gov/study/NCT04782271 (accessed on 16 December 2025).

47. ClinicalTrials.gov. Zilebesiran as Add-on Therapy in Patients With High Cardiovascular Risk and Hypertension Not Adequately Controlled by Standard of Care Antihypertensive Medications (KARDIA-3); available from: https://clinicaltrials.gov/study/NCT06272487 (accessed on 16 December 2025).

48. ClinicalTrials.gov. Evaluation of OLX10212 in Patients With Neovascular Age-related Macular Degeneration; available from: https://clinicaltrials.gov/study/NCT05643118 (accessed on 16 December 2025).

49. ClinicalTrials.gov. A Study to Evaluate ALN-HTT02 in Adult Patients With Huntington's Disease; available from: https://clinicaltrials.gov/study/NCT06585449 (accessed on 16 December 2025).

50. ClinicalTrials.gov. PBMC-based Leukocyte Immunotherapy (PALINDROM); available from: https://clinicaltrials.gov/study/NCT06172894 (accessed on 16 December 2025).

51. ClinicalTrials.gov. Olpasiran Trials of Cardiovascular Events And LipoproteiN(a) Reduction – DOSE Finding Study; available from: https://clinicaltrials.gov/study/NCT04270760 (accessed on 16 December 2025).

52. ClinicalTrials.gov. Study to Evaluate Safety, Tolerability, PK and PD of DCR-PHXC in PH Type 3 Patients (PHYOX4); available from: https://clinicaltrials.gov/study/NCT04555486 (accessed on 16 December 2025).
